# Can Parenting Practices Explain the Differences in Beverage Intake According to Socio-Economic Status: The Toybox-Study

**DOI:** 10.3390/nu8100591

**Published:** 2016-09-23

**Authors:** An-Sofie Pinket, Marieke De Craemer, Ilse De Bourdeaudhuij, Benedicte Deforche, Greet Cardon, Odysseas Androutsos, Berthold Koletzko, Luis A. Moreno, Piotr Socha, Violeta Iotova, Yannis Manios, Wendy Van Lippevelde

**Affiliations:** 1 Department of Public Health, Ghent University, Ghent 9000, Belgium; Benedicte.Deforche@UGent.be (B.D.); Wendy.VanLippevelde@UGent.be (W.V.L.); 2 Department of Movement and Sports Sciences, Ghent University, Ghent 9000, Belgium; Marieke.DeCraemer@UGent.be (M.D.C.); Ilse.DeBourdeaudhuij@UGent.be (I.D.B.); Greet.Cardon@UGent.be (G.C.); 3 Department of Human Biometry and Biomechanics, Vrije Universiteit Brussel, Brussels 1000, Belgium; 4 Department of Nutrition and Dietetics, School of Health Science and Education, Harokopio University, Athens 17671, Greece; oandrou@hua.gr (O.A.); manios.toybox@hua.gr (Y.M.); 5 Ludwig-Maximilians-University of Munich, Dr. von Hauner Children’s Hospital, München 80337, Germany; Berthold.Koletzko@med.uni-muenchen.de; 6 GENUD Research Group, University of Zaragoza, Zaragoza 50009, Spain; lmoreno@unizar.es; 7 The Children’s Memorial Health Institute, Warsaw 04-730, Poland; p.socha@czd.pl; 8 Department of Pediatrics, Medical University of Varna, Varna 9002, Bulgaria; iotova_v@yahoo.com

**Keywords:** socio-economic status inequalities, beverage choices, educational level, preschoolers, parents, water, soft drinks, prepacked fruit juice, parenting practices

## Abstract

Previous research indicated that preschoolers of lower socioeconomic status (SES) consume less healthy beverages than high SES preschoolers. The purpose of this study is to investigate the mediating role of parenting practices in the relationship between SES and plain water, soft drink and prepacked fruit juice (FJ) consumption in European preschoolers. Parents/caregivers of 3.5 to 5.5 years old (*n* = 6776) recruited through kindergartens in six European countries within the ToyBox-study completed questionnaires on socio-demographics, parenting practices and a food frequency questionnaire. Availability of sugared beverages and plain water, permissiveness towards sugared beverages and lack of self-efficacy showed a mediating effect on SES-differences in all three beverages. Rewarding with sugared beverages significantly mediated SES-differences for both plain water and prepacked FJ. Encouragement to drink plain water and awareness significantly mediated SES-differences for, respectively, plain water and prepacked FJ consumption. Avoiding negative modelling did not mediate any associations. Overall, lower SES preschoolers were more likely to be confronted with lower levels of favourable and higher levels of unfavourable parenting practices, which may lead to higher sugared beverage and lower plain water consumption. The current study highlights the importance of parenting practices in explaining the relation between SES and both healthy and unhealthy beverage consumption.

## 1. Introduction

A recent European study in preschoolers showed that only about half of water intake from beverages was derived from plain water (both tap and bottled water without any additives) and nearly a quarter of total water intake from beverages from sweetened beverages (such as soft drinks and especially prepacked fruit juice) [1]. The excessive intake of added sugars through these sweetened beverages can lead to an energy imbalance (i.e., energy intake that exceeds energy expenditure) and thus to overweight [2,3]. The importance of healthy choices already starts early in life since dietary habits are being formed at a young age and track into adolescence and adult life [4]. Therefore, developing healthy habits should already start in the first years of life.

Given the young age of preschool children, parents play a fundamental role in developing a home environment that stimulates healthy eating habits among their children through general parenting style (authoritative, authoritarian, permissive or neglectful) and parenting practices (such as availability and accessibility, role-modelling and rewarding) related to eating behaviours [5,6]. General parenting styles has been defined as a set of attitudes and beliefs that create an emotional climate and determines behavioural expression between parent and child. In contrast to general parenting styles, parenting practices are behaviour-specific acts of parenting which may differ across children within a family depending on children’s age, gender, eating and activity behaviour and weight status, and which are situation-specific [7].

Earlier studies found a significant influence of parenting practices on soft drink intake [8,9,10,11,12,13]. It is expected that, also in the consumption of other beverages, parenting practices play an important role but research in preschoolers on this topic is currently missing.

Previous research found that the home environment in lower socioeconomic status (SES) families is less supportive for a healthy lifestyle [14,15]. A study on beverage consumption in European preschool children found differences in beverage intake by socioeconomic status. Preschoolers of lower SES drank more sugared beverages and less plain water than their high SES peers [1]. De Coen et al. (2012) found similar SES-differences in soft drink consumption in Flemish preschoolers and revealed that this difference was mediated by parenting practices [9]. Similar mediation effects are expected for plain water and prepacked fruit juice consumption in other European countries, but cross-European research is currently missing. In order to limit the widening of health inequalities, it is important to specifically focus on reaching and changing the low SES households through health promotion initiatives. As such, investigating the mediating role of parenting practices on SES-differences in beverages choices is highly important. Therefore, the aim of the present study was to explore the mediating role of parenting practices in explaining differences in preschoolers’ plain water, soft drink and prepacked fruit juice consumption by socioeconomic status. Data were collected in the context of the ToyBox-study (Multifactorial evidence-based approach using behavioural models in understanding and promoting fun, healthy food, play and policy for the prevention of obesity in early childhood) in six European countries [16].

## 2. Materials and Methods

### 2.1. Study Background

The ToyBox-study is an EU-funded large-scale study of preschoolers (3.5–5.5 years old) and their families from six European countries (Belgium, Bulgaria, Germany, Greece, Poland, and Spain). It aimed to develop and evaluate a kindergarten-based, family-involved intervention to prevent overweight and obesity in preschool children [16]. For the present study, the baseline-data from the ToyBox-study were used.

The ToyBox-study was approved by Ethical Committees in all six European countries, in line with national regulations (i.e., the Ethical Committee of Ghent University Hospital (Belgium), Committee for the Ethics of the Scientific Studies (KENI) at the Medical University of Varna (Bulgaria), Ethikkommission der Ludwig-Maximilians-Universität München (Germany), the Ethics Committee of Harokopio University of Athens (Greece), Ethical Committee of Children’s Memorial Health Institute (Poland), and CEICA (Comité Ético de Investigación Clínica de Aragón (Spain)).

### 2.2. Participants

Preschool children between 3.5 and 5.5 years old were recruited from six European countries. These children and their families were recruited at kindergartens, daycare centres or preschool settings, depending on the country regulations and legislation. In order to avoid confusion for the reader, all these settings will be referred to as “kindergartens” in this paper. Kindergartens were recruited from different socio-demographic backgrounds within each of the provinces (West- and East-Flanders in Belgium, Varna in Bulgaria, Bavaria in Germany, Attica in Greece, Warsaw and surroundings in Poland, and Zaragoza in Spain).

A minimum sample of 800 children and their families and 20 kindergartens per country, resulting in a total sample of 4800 children and their families and 120 kindergartens, was initially targeted. However, in order to account for an estimated dropout rate of about 30%, a minimum total number of about 6500 children and their families were aimed to be recruited in the six participating countries [17]. Data collection occurred between May and June 2012. Parents/caregivers were asked for written consent for the participation of their child and themselves in the study. Only preschoolers whose parents/caregivers gave their consent were included in the study. Detailed sampling methods have been described elsewhere [17].

### 2.3. Measures

#### 2.3.1. Core Questionnaire

Parents/caregivers were asked to complete a core questionnaire, consisting of questions on socio-demographics and child and parent/caregiver behaviour (such as parenting practices).

##### Socio-Demographic Variables

Gender and date of birth were reported by one of the parents/caregivers of the child. Children’s age was computed based on the date of birth and the date when the questionnaire was completed. All questionnaires are available on the ToyBox-website (www.toybox-study.eu) and in the second ToyBox supplement issue [18]. Education of the parents/caregivers was also reported in the core questionnaire. The education level of the mother was used as an SES indicator. Educational level has been identified as an important indicator for SES and maternal education is often seen as more influencing for the child than education of the father given that mothers are often the primary caregiver [9,19,20]. The education level was dichotomized into lower (14 or fewer years of education) and high (more than 14 years of education) SES, similar to the SES measure used in the large-scale European ENERGY-study, which distinguishes families with a mother who has completed medium or higher education, college or university training from other families [21].

##### Parenting Practices

Seventeen statements regarding plain water, soft drinks and prepacked fruit juice consumption of their preschool child were presented, of which twelve statements, focusing on parenting practices, were used in the current study. The development of the questions included in the parental questionnaire was based on questionnaires previously used in large European studies of which construct validity of the items on multiple energy balance-related behaviours, their potential determinants, and parenting practices has been shown to be good [22]. In addition, in the study of González-Gil et al. (2014), a moderate to good test-retest reliability (ICC ranged from 0.409 to 0.693) for all questions on parenting practices used in this study was found [23].

Parents/caregivers were asked to indicate what was most appropriate for them, choosing from five Likert-type answer categories (strongly disagree, disagree, neither agree nor disagree, agree and strongly agree). An exploratory factor analysis of principal components was executed to investigate the possibility to form subscales based on the twelve parental items regarding plain water, soft drinks and prepacked fruit juice consumption of their preschool child. However, given the differences in content of the items loading on the retained factors, it was decided to combine only some of the items into subscales, based on previous literature [8,9]. Components consisting of several items were tested for cohesion with Cronbach’s alpha (Table 1). Next, the items of one component were summed. In this way, eight parenting practice components were constructed: “Availability of soft drinks/prepacked juices”, “Availability of plain water”, “Permissiveness”, “Avoiding negative modelling”, “Awareness”, “Encouragement”, “Rewarding” and “Self-efficacy”. Table 1 shows the exact formulation of the questionnaire items and the psychometric characteristics, as well as the eight constructed parenting practices.

Preschoolers with missing data on all parenting practice questions were excluded from the study (*n* = 233). The excluded preschoolers did not differ significantly from included preschoolers in terms of SES or intake.

#### 2.3.2. Food Frequency Questionnaire

Next to completing the core questionnaire, parents/caregivers were asked to describe the child’s usual food and beverage habits over the last 12 months in a food frequency questionnaire (FFQ) for young children, based on a previously validated FFQ developed by Huybrechts et al. [24]. The FFQ of Huybrechts and colleagues was developed to assess preschool children’s food and beverage group estimates and consumption patterns. It has been validated in general (i.e., for all food items included in the FFQ) and this includes the various types of beverages covered in the FFQ and used in this study. Results of the validation of the FFQ by Huybrechts and colleagues showed moderate to good reproducibility (intra class correlation (ICC) ranged from 0.62 to 0.79) and good relative validity (Spearman correlation ranged from 0.56 to 0.65) for beverages [24]. The relative validity of food intake estimates derived from the FFQ was evaluated through comparison with a reference method, being a 3-day estimated dietary record (EDR), collected about one week after the collection of the completed FFQ [24].

The selected beverages used in this study were plain water (both tap and bottled water), soft drinks and other fruit juice (pre-packed/bottled fruit juice). For each of these beverages, the frequency of consumption was asked. Response categories were: “never or less than once per month”, “1–3 days per month”, “1 day per week”, “2–4 days per week”, “5–6 days per week” and “every day”. Next, the average consumption per day was asked. The response categories were “100 mL or less”, “100–200 mL”, “200–300 mL”, “300–400 mL”, “400–500 mL”, “500–600 mL”, “600–700 mL”, “700–800 mL”, “800–900 mL”, “900–1000 mL” and “1000 mL or more”. From these data, the average amount of the different beverages in millilitres per day was calculated by multiplication of number of days per week and intake per day in millilitres divided by 7.

Preschool children who had a valid measurement (i.e., fitting the answering categories from which parents/caregivers could choose) for frequency and portion size for at least one beverage were included. This means that preschoolers who had no valid data (both on frequency and portion size) on all three beverages were excluded from the study (*n* = 51).

### 2.4. Statistical Analyses

**Figure 1:**
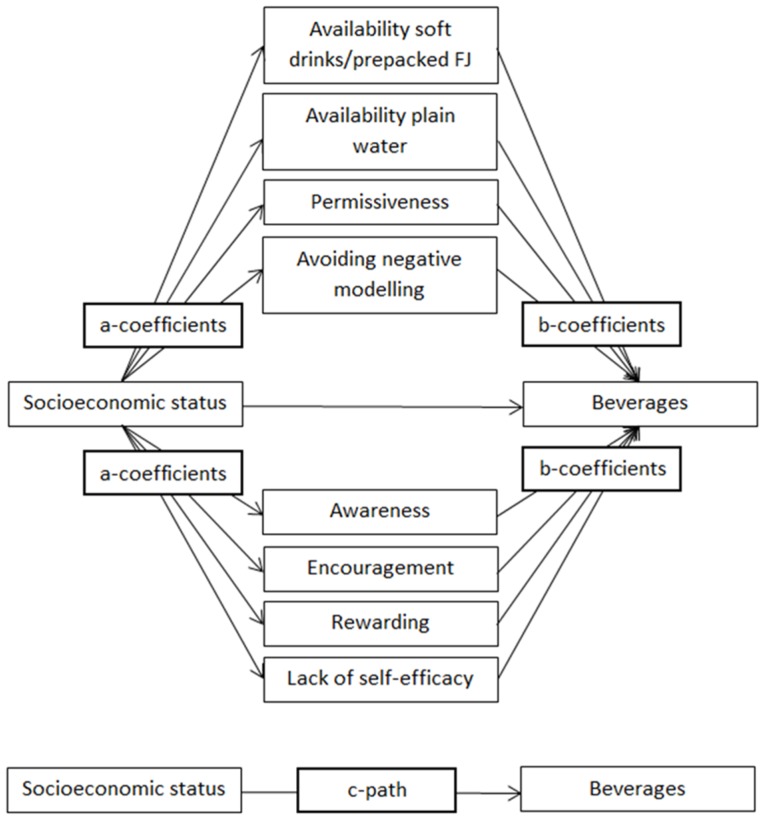
Mediation model of the association between socioeconomic status, parenting practices and beverage intake.

First, descriptive statistics were performed using IBM SPSS Statistics for Windows, version 21.0 (IBM Corp, Amonk, NY, USA, 2012). Next, multilevel analyses were performed using MLWiN, version 2.30 (Centre for Multilevel Modelling, University of Bristol, Bristol, UK, 2014) to take clustering of preschool children in kindergarten classes, and of kindergartens classes in kindergartens into account (a three level structure: preschooler within kindergarten class within kindergarten, was used). Before building a multiple multilevel mediation model, the mediation analyses were conducted for the single mediators. Since all parenting practices were significant single mediators for at least one of the beverages, they were all entered in one model (Figure 1), resulting in a multiple multilevel mediating model. Differences were tested in the total sample and in all six country-specific samples. To perform analysis in the total sample, country was added as a fourth level. Significance level was set at *p* < 0.05.

The mediating role of parenting practices on the association between SES and beverage intake was tested using the product-of-coefficient test of MacKinnon [25]. This test consists of different stages: (1) the action theory test which estimates the association between SES and the potential mediators (a-coefficients); (2) conceptual theory test which estimate the association between the potential mediators and beverage intakes (b-coefficients) controlling for the independent variable (i.e., SES); (3) the calculation of the product of the two coefficients (ab), representing the mediating effect; and (4) the calculation of dividing ab by its standard error (SE) to assess the statistical significance of the mediating role. To calculate the SE, the Sobel test for multiple mediation was used. According to MacKinnon, a significant total association (c-path) is not necessary for mediation to occur [25]. The existence of mediation in the absence of a total association might be due to unmeasured variables that suppress the association between SES and fruit juice intake.

The analysis was adjusted for age and gender of the preschool child. Three multiple mediating models were conducted, one for each beverage intake (plain water, soft drinks and prepacked fruit juice). Significance level was set at *p* < 0.05.

## 3. Results

### 3.1. Population Characteristics

Table 2 presents the characteristics for the total sample and for each country separately. The total sample included 6776 preschoolers (mean age 4.8 ± 0.4 years, 52.1% boys) from six European countries, 39.8% had a mother with a lower level of education (≤14 years of education). In addition, preschoolers’ daily intake of plain water, soft drinks and prepacked fruit juice is presented in Table 2, as well as mean scores and standard deviation on the eight parenting practices included in the study.

### 3.2. Mediation Analyses on the Country-Specific Samples (See also Table 3)

#### 3.2.1. Associations between SES and Beverage Intake (c-Path)

A significant difference was found between lower and high SES preschoolers for plain water (*c* = 26.558, *SE* = 8.634, *p* < 0.001) and soft drink intake (*c* = 24.791, *SE* = 3.683, *p* < 0.001), not for fruit juice. Lower SES preschoolers consume less plain water and more soft drinks than their high SES peers.

#### 3.2.2. Associations between SES and Potential Mediators (a-Coefficients)

Significant SES-differences were found for all parenting practices. Higher SES was associated with less availability of soft drinks/prepacked fruit juice, higher availability of plain water, less permissiveness towards sugared beverages, more avoiding of negative modeling, more awareness of the negative advice on daily soft drink/prepacked fruit juice consumption, more encouragement to drink plain water, less rewarding with sugared beverages and higher levels of self-efficacy towards persistency to refuse sugared beverage consumption despite the will of their child compared to the lower SES preschoolers.

#### 3.2.3. Associations between Potential Mediators and Beverage Intake Controlled for SES (b-Coefficients)

##### Plain Water

All parenting practices, except for avoiding negative modelling and awareness of the negative advice on daily soft drink/prepacked fruit juice consumption, were significantly and independently associated with plain water consumption. Higher availability of plain water, more encouragement to drink plain water and more rewarding with sugared beverages was associated with higher plain water consumption, while higher availability of sugared beverages, more permissiveness toward sugared beverages and a lack of self-efficacy towards persistency to refuse sugared beverage consumption despite the will of their child was associated with lower plain water intake.

##### Soft Drinks

Avoiding negative modelling, awareness of the negative advice on daily soft drink/prepacked fruit juice consumption, encouragement to drink plain water and rewarding with sugared beverages were significantly and independently associated with soft drink consumption. Higher availability of sugared beverages, more permissiveness towards sugared beverages and a lack of self-efficacy towards persistency to refuse sugared beverage consumption despite the will of their child were associated with higher soft drink intake, while a higher availability of plain water was associated with lower intakes of soft drinks.

##### Prepacked Fruit Juice

All parenting practices, except for avoiding negative modelling and encouragement to drink plain water, were significantly associated with prepacked fruit juice consumption. Higher availability of sugared beverages, more permissiveness towards sugared beverages and a lack of self-efficacy towards persistency to refuse sugared beverage consumption despite the will of their child was associated with a higher consumption of prepacked fruit juices, while higher availability of plain water, less awareness of the negative advice on daily soft drink/prepacked fruit juice consumption and more rewarding with sugared beverages was associated with lower intakes of prepacked fruit juice.

#### 3.2.4. Mediating Effect of Parenting Practices on the Associations between SES and Beverage Intake (ab-Coefficients)

Availability of soft drinks/prepacked fruit juice, availability of plain water, permissiveness towards sugared beverages and lack of self-efficacy towards persistency to refuse sugared beverage consumption despite the will of their child showed a mediating effect on the relation between SES and plain water (proportion mediated, respectively: 42.5%, 29.0%, 15.4% and 17.3%), soft drinks (proportion mediated, respectively: 18.1%, 6.5%, 15.0% and 4.0%) and prepacked fruit juice consumption (non-significant association between SES and prepacked fruit juice, so proportion mediation not shown). Rewarding with sugared beverages significantly mediated (i.e., suppressed) the association between SES and both plain water and prepacked fruit juice consumption for −6.6% and −19.5% respectively. Encouragement to drink plain water showed a mediating effect on the relation between SES and plain water consumption (12.1%). Awareness of the negative advice on daily soft drink/prepacked fruit juice consumption significantly mediated the association between SES and prepacked fruit juice consumption for 45.3%. Avoiding negative modelling did not significantly mediate any associations, as the conceptual theory was not significant.

### 3.3. Mediation Analyses on the Country-Specific Samples (See also Table 4, the Most Important Results Are Discussed Below)

#### 3.3.1. Associations between SES and Beverage Intake (c-Path)

For Belgium and Germany, significant associations for plain water and soft drinks with SES were found. For the Greek and Polish sample, respectively, associations with SES were found only for plain water and soft drinks. For Bulgaria and Spain, no significant associations between SES and beverage intake were found.

#### 3.3.2. Associations between SES and Potential Mediators (a-Coefficients)

In the Belgian sample, SES-differences were found for all eight parenting practices. In Bulgarian preschoolers, SES differences were found for availability of soft drinks/prepacked fruit juice, permissiveness towards sugared beverages, rewarding with sugared beverages and lack of self-efficacy towards persistency to refuse sugared beverage consumption despite the will of their child. In the German sample, the a-path was significant for availability of both soft drinks/prepacked fruit juice and plain water, permissiveness towards sugared beverages, avoiding negative modelling and rewarding with sugared beverages. In Greek preschoolers, SES-differences were found for all parenting practices, except for encouragement to drink plain water. In the Polish sample, the a-path was significant for all parenting practices, except for lack of self-efficacy towards persistency to refuse sugared beverage consumption despite the will of their child. In Spanish preschool children, only for availability of soft drinks/prepacked fruit juice, permissiveness towards sugared beverages and awareness of the negative advice on daily soft drink/prepacked fruit juice consumption SES-differences were found. More details can be found in Table 4.

#### 3.3.3. Associations between Potential Mediators and Beverage Intake (b-Coefficients)

The b-path differed in all six countries. Results can be found in Table 4.

#### 3.3.4. Mediating Effect of Parenting Practices on the Associations between SES and Beverage Intake (ab-Coefficients)

Availability of soft drinks/prepacked fruit juice was a significant mediator of the relationship between SES and intakes of (almost) all beverages in all six countries. Awareness of the negative advice on daily soft drink/prepacked fruit juice consumption mediated the relation between SES and soft drink intake in all countries, except for Bulgaria and Greece. Permissiveness towards sugared beverages was a significant mediator of the relation between SES and soft drink consumption in Belgian, German and Polish preschoolers. Other mediated effects in the country-specific samples can be found in Table 4.

## 4. Discussion

The aim of the current study was to identify the mediating role of parenting practices on SES-differences in plain water, soft drink and prepacked fruit juice consumption. Since an unhealthy beverage intake was already found at preschool age and lower SES preschoolers were found to make unhealthier beverage choices than their high SES peers [1], it is important to investigate the underlying mechanisms of these SES-differences in intake to avoid the widening of health inequalities.

A previous paper on the ToyBox-study already showed that lower SES preschoolers consume less plain water and more soft drinks than their high SES peers, no differences were found for prepacked fruit juice [1]. A possible explanation for the lack of SES-differences for prepacked fruit juice could be found in a misperception of the sugar content of prepacked fruit juices both in lower and highly educated parents, in contrast to perceptions of soft drinks which are more accurate in highly educated parents. Results of the current study revealed that in all included European countries, lower SES mothers reported lower levels of favourable parenting practices (making plain water available, avoiding negative modelling, awareness of the negative advice on daily soft drink/prepacked fruit juice consumption and encouragement to drink plain water) and higher levels of unfavourable parenting practices (making soft drinks/prepacked fruit juice available, permissiveness towards sugared beverages, rewarding with sugared beverages and lack of self-efficacy towards persistency to refuse sugared beverage consumption despite the will of their child) compared to higher SES mothers. In a study on Flemish preschoolers, comparable results were found for permissiveness and avoiding negative modelling [26]. However, studies investigating SES-differences in beverage parenting practices in preschoolers’ parents are scarce. The current study indicates that lowers SES mothers apply less desirable parenting practices regarding their preschoolers’ beverage intake, than high SES mothers. Similar results were found in the country-specific samples.

Consistent with previous studies, both in preschoolers and older children [8,9,10,26], higher levels of availability of soft drinks/prepacked fruit juice, permissiveness towards sugared beverages and lack of self-efficacy towards persistency to refuse sugared beverage consumption despite the will of their child were associated with less healthy beverage consumption in preschoolers (less plain water and more soft drink and prepacked fruit juice consumption). In addition, a strong potential impact of making plain water available at home was found, as it was associated with positive beverage choices in preschoolers (higher consumption of plain water and lower consumption of soft drinks and prepacked fruit juice). In addition, higher levels of awareness of the negative advice about daily consumption of sugared beverages were associated with healthier beverage choices (less prepacked fruit juice consumption). Higher levels of rewarding with sugared beverages were also associated with healthy beverage consumption (more plain water and less prepacked fruit juice consumption). A possible explanation for this unexpected finding could be that preschoolers who usually drink plain water and are only occasionally allowed to drink sugared beverages are more likely to get sugared beverages as a reward, whereas preschoolers who already consume a lot of sugared beverages, do not consider sugared beverages as a reward anymore. In the current study, no association was found between avoiding negative modelling and soft drink consumption. Overall, it can be concluded that more preferable parenting practices are associated with healthier beverage choices and less preferable parenting practices were associated with less healthy beverage choices.

The current study showed that SES-inequalities in beverage intake were partly mediated by certain parenting practices. Availability of soft drinks/prepacked fruit juice, availability of plain water, permissiveness towards sugared beverages and lack of self-efficacy to refuse sugared beverage consumption mediated the relation between SES and all three beverages. This suggests that preschoolers of lower SES backgrounds were more likely to be confronted with higher availability of soft drinks/prepacked fruit juices, lower availability of plain water, higher levels of permissiveness towards sugared beverages and a lack of self-efficacy of their parents compared to preschoolers of high SES backgrounds, which may result both in higher sugared beverage (soft drinks and prepacked fruit juices) and lower healthy beverage (plain water) consumption. Nevertheless, reverse causality cannot be ruled out due to the cross-sectional nature of the study. Avoiding negative modelling was the only non-significant mediator in all three beverages, suggesting that this parenting practice plays no important role in explaining SES-differences in preschoolers’ beverage intake. In addition, in the study on soft drinks in Flemish preschoolers of De Coen and colleagues (2012) [9], availability of soft drinks and permissiveness towards sugared beverages were found as important mediators, while avoiding negative modelling was also found as a non-significant mediator in the relation between SES and soft drinks. Awareness of the negative advice on daily soft drink/prepacked fruit juice consumption only mediated SES-inequalities in prepacked fruit juice consumption, not in plain water and soft drink consumption. This could suggest that lower SES preschoolers were more likely to have parents who are not aware of the recommendation to limit sugared beverage intake, only resulting in higher consumption of prepacked fruit juice consumption. A possible explanation could be found in the fact that awareness about plain water and soft drink consumption is higher than awareness about prepacked fruit juice consumption, resulting in a lower impact of awareness on SES-differences in plain water and soft drink consumption compared to prepacked fruit juice consumption. Parents are already aware of the importance of consuming plain water and avoiding soft drink intake in a healthy diet, but are not aware of the high sugar level in prepacked fruit juice [8]. Furthermore, encouragement to drink plain water was only a significant mediator explaining differences in plain water consumption. This suggests that lower SES preschoolers were less likely to be encouraged to drink plain water by their mothers than high SES preschoolers, which may result in lower plain water consumption in lower SES preschoolers, not higher sugared beverage intake. Rewarding with sugared beverages mediated the relation between SES and plain water and prepacked fruit juice consumption, but not soft drink intake. To limit the widening of health inequalities, availability at home seems to be most important parenting practice to work on, both in healthy and unhealthy beverages, followed by increasing self-efficacy to be persistent in refusing sugared beverage consumption and decreasing permissiveness towards sugared beverages. In addition, in older children (Dutch 11 years old), availability of soft drinks was found as an important mediator in the relation between maternal education and soft drinks consumption [27]. Although SES as such cannot be altered, parenting practices can be modified. It is crucial for lower SES parents to master the parenting practices that are found to be important to tackle SES-inequalities in preschoolers’ beverage intake, more specifically availability of both healthy and unhealthy beverages, permissiveness and lack of self-efficacy for all three beverages, rewarding for plain water and prepacked fruit juice, encouragement for plain water and awareness for prepacked fruit juice. Thus, targeting the home environment in lower SES households should already start at young age in order to prevent health inequalities at later age.

Mixed results were found in the country-specific samples. Nevertheless, availability of soft drinks/prepacked fruit juice was found to be the most univocal mediator of the association between SES and beverage intake, which underlines the importance of advocating limiting the availability of sugared beverages, especially at lower SES parents’ homes in order to tackle inequalities in beverage intake by SES across Europe. Similar results were found in a study among 10 to 12 years old of eight European countries, where availability was also found as the most persistent parenting practice after stratification to explain the relation between SES inequalities and soft drink/prepacked fruit juice consumption [26]. In addition, the HBSC study, a large-scale study investigating time trends and correlates of soft drinks in twenty-four European countries, found that the availability of soft drinks is steadily and significantly increasing especially in lower SES households [28]. Consequently, decreasing the availability of unhealthy drinks in all European households with a focus on lower SES families is essential. Since literature on parental practices as a possible mediator in the association between SES and beverage intake in preschoolers is scarce, future studies should investigate the mediating role of parenting practices in the relationship between SES and both healthy and unhealthy beverages in preschoolers in more detail, for instance by including more parenting practices and more beverages (such as plain and sugared milk) and by using longitudinal data. In addition, future intervention studies should study the effect of changing parenting practices on beverage intake in preschool children.

Limitations of the current study should be acknowledged. The data are self-reported which may result in social desirability. However, this was partially covered by ensuring anonymity. The cross-sectional nature of the design does not permit the examination of causality of the investigated relationships. We acknowledge that the ToyBox-sample is not a fully representative European sample, due to sampling in specific regions in each country. Samples included preschoolers of low, medium and high SES backgrounds and in each kindergarten (almost) complete classes were included. The samples can give a fair approximation of the average situation in each country. The procedure of sampling in specific regions has also been used in several other European studies such as HELENA and ENERGY [29,30]. Rewarding was operationalized in a single-item question asking for both instrumental and emotional feeding. However, instrumental and emotional feeding are not always related. It would be interesting to study this parenting practice in more detail by looking into differences between using food in between meals to regulate a child’s emotions (emotional feeding) and using food as a reward or withholding food as a punishment (instrumental feeding) [31]. Awareness and lack of self-efficacy may be regarded as precursors of parenting practices instead of actual parental behaviours. Future research should study these factors as potential precursors of the actual behaviour, which can lead to interesting findings.

A major strength of this study is the large sample of preschoolers from six European countries and the standardized data collection protocol across the different countries. In addition, to our knowledge, this is the first study that examined the mediating role of eight different parenting practices explaining differences in both European preschoolers’ healthy and unhealthy beverage consumption by socioeconomic status.

## 5. Conclusions

The current study highlighted the importance of parenting practices, especially availability of healthy and unhealthy beverages at home, permissiveness towards sugared beverages and lack of self-efficacy to refuse sugared beverage consumption, in explaining the relation between SES and consumption of both healthy and unhealthy beverages. Teaching low SES parents to make healthy beverages available at home, to decrease the availability of sugared beverages at home, to set rules regarding sugared beverages consumption at home and to increase their self-efficacy to maintain these rules might lower the gap in healthy beverage consumption of preschoolers of different SES backgrounds.

## Figures and Tables

**Table 1:** Formulations of the questionnaire items and the psychometric characteristics.

Factor	Question Item	Answer Categories
Availability soft drinks/prepacked fruit juice (Cronbach α = 0.68)	I make soft drinks or prepacked juices always available for my child. During meals, soft drinks or prepacked juices are always available on the table.	1 = strongly disagree– 5 = strongly agree
Availability plain water (Cronbach α = 0.41)	I make water always available for my child. During meals, water is always available on the table.	1 = strongly disagree– 5 = strongly agree
Permissiveness towards soft drinks/prepacked fruit juice (Cronbach α = 0.62)	My child is allowed to drink soft drinks or prepacked juices as much as he/she likes. My child can drink soft drinks or prepacked fruit juices whenever he/she asks for.	1 = strongly disagree– 5 = strongly agree
Avoiding negative modelling	If I would like to drink soft drinks or prepacked juices, I would try to restrain myself because of the presence of my child.	1 = strongly disagree– 5 = strongly agree
Awareness (Cronbach α = 0.60)	It is bad for my child to drink soft drinks every day. It is bad for my child to drink prepacked fruit juices every day.	1 = strongly disagree– 5 = strongly agree
Encouragement	I encourage my child to drink water.	1 = strongly disagree– 5 = strongly agree
Rewarding	I give soft drinks or prepacked juices to my child as a reward or to comfort him/her.	1 = strongly disagree– 5 = strongly agree
Lack of self-efficacy	I find it difficult to give my child water if he/she wants soft drinks or prepacked juices.	1 = strongly disagree– 5 = strongly agree

**Table 2:** Characteristics of participants of the total sample and each country separately.

	Total	Belgium	Bulgaria	Germany	Greece	Poland	Spain
N	6776	917	752	1139	1733	1384	851
Age	4.8 ± 0.4	4.4 ± 0.5	4.9 ± 0.3	4.5 ± 0.5	4.9 ± 0.3	4.9 ± 0.3	4.9 ± 0.3
Gender (% male)	52.1%	52.2%	50.5%	51.9%	50.8%	53.0%	54.8%
SES *, % lower SES (=≤14 years of education)	39.8%	34.4%	40.8%	51.6%	51.3%	21.0%	36.1%
Ethnicity (One or both parents are not born in the country of residence)	16%	12.4%	3.5%	29.9%	24.4%	3.7%	15.6%
Plain water intake (mL/day)	546	414	658	490	629	394	739
Soft drink intake (mL/day)	56	61	35	42	13	155	14
Prepacked FJ intake (mL/day)	104	93	99	103	70	174	75
Availability soft drinks/prepacked FJ [1,2,3,4,5,6,7,8,9,10]	3.8 ± 1.8	3.9 ± 1.9	4.0 ± 1.9	3.9 ± 1.9	3.4 ± 1.4	4.2 ± 1.8	3.5 ± 1.5
Availability plain water [1,2,3,4,5,6,7,8,9,10]	8.9 ± 1.4	9.1 ± 1.3	8.4 ± 1.5	9.0 ± 1.3	9.1 ± 1.1	8.2 ± 1.6	9.5 ± 1.0
Permissiveness [1,2,3,4,5,6,7,8,9,10]	3.9 ± 1.7	4.1 ± 1.6	4.2 ± 1.9	3.7 ± 1.8	3.4 ± 1.2	4.1 ± 1.8	4.0 ± 1.6
Avoiding negative modelling [1,2,3,4,5]	3.5 ± 1.1	3.2 ± 1.1	3.3 ± 1.2	3.8 ± 1.1	4.0 ± 1.0	3.2 ± 1.1	3.3 ± 1.1
Awareness [1,2,3,4,5,6,7,8,9,10]	8.1 ± 1.9	8.1 ± 1.7	7.7 ± 2.2	8.1 ± 1.8	8.5 ± 1.7	8.0 ± 1.8	7.8 ± 1.9
Encouragement [1,2,3,4,5]	4.5 ± 0.7	4.5 ± 0.6	4.5 ± 0.8	4.4 ± 0.8	4.5 ± 0.7	4.5 ± 0.8	4.6 ± 0.7
Rewarding [1,2,3,4,5]	1.6 ± 0.8	1.6 ± 0.8	1.7 ± 0.9	1.5 ± 0.8	1.5 ± 0.8	1.8 ± 0.9	1.5 ± 0.8
Lack of self-efficacy [1,2,3,4,5]	2.3 ± 1.2	2.0 ± 1.0	2.1 ± 1.1	2.5 ± 1.2	1.9 ± 1.0	3.1 ± 1.2	1.8 ± 1.0

* Years of school education mother.

**Table 3:** Results from the multilevel multiple regression analyses (four level random intercept model) with plain water, soft drinks and prepacked fruit juice as the outcome variables. All analyses were adjusted for preschoolers’ age and gender (total sample).

Parenting Practices	a ^†^ (SE)	b ^‡^ (SE)	ab ^§^ (SE)	95% CI of ab	% Mediated Effect ^|^
**Plain water**					
Availability soft drinks/prepacked FJ	**−0.541 (0.050)**	**−20.888 (3.014)**	11.300 (1.936)	**7.505 to 15.096**	42.5
Availability plain water	**0.177 (0.038)**	**43.443 (3.228)**	7.689 (1.747)	**4.265 to 11.113**	29.0
Permissiveness	**−0.430 (0.047)**	**−9.496 (3.103)**	4.083 (1.407)	**1.326 to 6.841**	15.4
Avoiding negative modelling	**0.185 (0.032)**	−7.407 (3.839)	−1.370 (0.749)	−2.838 to 0.097	-
Awareness	**0.430 (0.053)**	−2.111 (2.288)	−0.908 (0.990)	−2.848 to 1.033	-
Encouragement	**0.080 (0.021)**	**40.167 (5.758)**	3.213 (0.961)	**1.330 to 5.097**	12.1
Rewarding	**−0.129 (0.024)**	**13.527 (4.865)**	−1.745 (0.707)	**−3.130 to −0.360**	−6.6
Lack of self-efficacy	**−0.139 (0.031)**	**−32.995 (3.790)**	4.586 (1.151)	**2.331 to 6.841**	17.3
Total	-	-	26.850 (2.817)	**21.329 to 32.370**	101.1
**Soft drinks**					
Availability soft drinks/prepacked FJ	**−0.541 (0.050)**	**8.297 (1.325)**	−4.489 (0.828)	**−6.112 to −2.865**	18.1
Availability plain water	**0.177 (0.038)**	**−9.164 (1.418)**	−1.622 (0.429)	**−2.463 to −0.781**	6.5
Permissiveness	**−0.430 (0.047)**	**8.643 (1.364)**	−3.716 (0.713)	**−5.115 to −2.318**	15.0
Avoiding negative modelling	**0.185 (0.032)**	−2.562 (1.688)	−0.474 (0.323)	−1.107 to 0.159	-
Awareness	**0.430 (0.053)**	−0.461 (1.004)	−0.198 (0.432)	−1.046 to 0.649	-
Encouragement	**0.080 (0.021)**	−0.684 (2.532)	−0.055 (0.203)	−0.453 to 0.343	-
Rewarding	**−0.129 (0.024)**	1.822 (2.139)	−0.235 (0.279)	−0.783 to 0.313	-
Lack of self-efficacy	**−0.139 (0.031)**	**7.199 (1.662)**	−1.001 (0.321)	**−1.630 to −0.371**	4.0
Total	-	-	−11.790 (0.955)	**−13.662 to −9.918**	47.6
**Prepacked FJ**					
Availability soft drinks/prepacked FJ	**−0.541 (0.050)**	**16.755 (1.461)**	−9.064 (1.152)	**−11.322 to −6.807**	124.0
Availability plain water	**0.177 (0.038)**	**−4.623 (1.563)**	−0.818 (0.328)	**−1.461 to −0.176**	11.2
Permissiveness	**−0.430 (0.047)**	**8.222 (1.504)**	−3.535 (0.753)	**−5.012 to −2.059**	48.4
Avoiding negative modelling	**0.185 (0.032)**	1.709 (1.860)	0.316 (0.348)	−0.367 to 0.999	-
Awareness	**0.430 (0.053)**	**−7.705 (1.108)**	−3.313 (0.628)	**−4.543 to −2.083**	45.3
Encouragement	**0.080 (0.021)**	−1.663 (2.791)	−0.133 (0.226)	−0.576 to 0.310	-
Rewarding	**−0.129 (0.024)**	**−11.066 (2.358)**	1.428 (0.404)	**0.636 to 2.219**	−19.5
Lack of self-efficacy	**−0.139 (0.031)**	**6.776 (1.833)**	−0.942 (0.330)	**−1.589 to −0.295**	12.9
Total	-	-	−16.063 (1.278)	**−18.567 to −13.558**	219.7

SE = standard error, CI = confidence interval, FJ = fruit juice; All significant associations are presented in bold font; ^†^ a-coefficients: Associations between SES (socioeconomic status) and potential mediators; ^‡^ b-coefficients: Associations between potential mediators (parenting practices) and beverage intake controlled for SES; ^§^ ab-coefficients: Mediating effect of parenting practices on the associations between SES and beverage intake; ^|^ Percentage mediated effect was not calculated if the mediation effect was not significant.

**Table 4:** Results from the multilevel multiple regression analyses (three level random intercept model) with plain water, soft drinks and prepacked fruit juice as the outcome variables. All analyses were adjusted for preschoolers’ age and gender (country-specific samples).

	**Belgium**					**Bulgaria**				
	**(*n* = 917)**					**(*n* = 752)**				
**Parenting Practices**	**a ^†^ (SE)**	**b ^‡^ (SE)**	**ab ^§^ (SE)**	**95% CI of ab**	**% Mediated Effect ^|^**	**a ^†^ (SE)**	**b ^‡^ (SE)**	**ab ^§^ (SE)**	**95% CI of ab**	**% Mediated Effect ^|^**
**Plain water**										
Availability soft drinks/prepacked FJ	**−1.110 (0.139)**	**−14.708 (6.012)**	16.326 (6.979)	**2.646 to 30.006**	33.7	**−0.450 (0.147)**	−2.952 (8.585)	1.328 (3.888)	−6.291 to 8.948	-
Availability plain water	**0.380 (0.096)**	**17.284 (7.667)**	6.568 (3.353)	−0.004 to 13.139	-	−0.073 (0.119)	**34.688 (7.914)**	−2.532 (4.168)	−10.702 to 5.637	-
Permissiveness	**−0.742 (0.120)**	−4.837 (6.727)	3.589 (5.025)	−6.260 to 13.438	-	**−0.323 (0.145)**	0.255 (8.434)	−0.082 (2.724)	−5.422 to 5.258	-
Avoiding negative modelling	**0.232 (0.087)**	**−19.683 (7.619)**	−4.566 (2.461)	−9.390 to 0.257	-	0.040 (0.095)	16.252 (10.676)	0.650 (1.602)	−2.490 to 3.790	-
Awareness	**0.792 (0.127)**	7.243 (5.619)	5.736 (4.544)	−3.170 to 14.643	-	−0.059 (0.175)	−5.934 (5.934)	0.350 (1.096)	−1.798 to 2.498	-
Encouragement	**0.193 (0.049)**	**80.420 (15.585)**	15.521 (4.957)	**5.805 to 25.238**	32.1	0.040 (0.064)	17.492 (14.739)	0.700 (1.265)	−1.780 to 3.180	-
Rewarding	**−0.184 (0.059)**	−11.023 (10.872)	2.028 (2.104)	−2.095 to 6.151	-	**−0.200 (0.069)**	3.330 (13.625)	−0.666 (2.735)	−6.026 to 4.694	-
Lack of self-efficacy	**−0.383 (0.077)**	**−23.291 (9.248)**	8.920 (3.970)	**1.139 to 16.702**	18.4	**−0.185 (0.085)**	**−45.731 (10.715)**	8.460 (4.636)	−0.092 to 17.012	-
Total	**-**	-	54.123 (7.700)	**39.031 to 69.214**	111.8	-	-	8.208 (7.135)	−5.776 to 22.192	-
Availability soft drinks/prepacked FJ	**−1.110 (0.139)**	**9.252 (2.784)**	−10.270 (3.347)	**−16.830 to −3.709**	20.7	**−0.450 (0.147)**	**5.707 (2.352)**	−2.568 (1.351)	−5.215 to 0.079	-
Availability plain water	**0.380 (0.096)**	−4.340 (3.554)	−1.649 (1.413)	−4.419 to 1.121	-	−0.073 (0.119)	0.330 (2.170)	−0.024 (0.163)	−0.344 to 0.296	-
Permissiveness	**−0.742 (0.120)**	**9.253 (3.114)**	−6.866 (2.564)	**−11.890 to −1.841**	13.8	**−0.323 (0.145)**	**6.583 (2.317)**	−2.126 (1.213)	−4.504 to 0.251	-
Avoiding negative modelling	**0.232 (0.087)**	−5.740 (3.538)	−1.332 (0.961)	−3.215 to 0.551	-	0.040 (0.095)	2.024 (2.932)	0.081 (0.225)	−0.360 to 0.522	-
Awareness	**0.792 (0.127)**	**−10.105 (2.601)**	−8.003 (2.427)	**−12.760 to −3.246**	16.1	−0.059 (0.175)	−1.623 (1.480)	0.096 (0.297)	−0.487 to 0.678	-
Encouragement	**0.193 (0.049)**	−13.970 (7.214)	−2.696 (1.551)	−5.737 to 0.345	-	0.040 (0.064)	5.115 (4.063)	0.205 (0.365)	−0.512 to 0.921	-
Rewarding	**−0.184 (0.059)**	−1.199 (5.043)	0.221 (0.931)	−1.603 to 2.045	-	**−0.200 (0.069)**	1.791 (3.748)	−0.358 (0.760)	−1.847 to 1.131	-
Lack of self-efficacy	**−0.383 (0.077)**	5.390 (4.276)	−2.064 (1.689)	−5.376 to 1.247	-	**−0.185 (0.085)**	**10.252 (2.945)**	−1.897 (1.028)	−3.911 to 0.118	-
Total	**-**	-	−32.659 (3.435)	**−39.392 to −25.927**	65.7	-	-	−6.592 (1.901)	**−10.318 to −2.866**	62.8
**Prepacked FJ**										
Availability soft drinks/prepacked FJ	**−1.110 (0.139)**	**7.004 (3.430)**	−7.774 (3.930)	**−15.477 to −0.072**	97.5	**−0.450 (0.147)**	**11.377 (3.503)**	−5.120 (2.298)	**−9.624 to −0.615**	−50.3
Availability plain water	**0.380 (0.096)**	−7.547 (4.379)	−2.868 (1.815)	−6.425 to 0.689	-	−0.073 (0.119)	−1.511 (3.232)	0.110 (0.297)	−0.471 to 0.692	-
Permissiveness	**−0.742 (0.120)**	5.662 (3.845)	−4.201 (2.933)	−9.946 to 1.547	-	**−0.323 (0.145)**	**10.896 (3.450)**	−3.519 (1.933)	−7.309 to 0.270	-
Avoiding negative modelling	**0.232 (0.087)**	−8.341 (4.349)	−1.935 (1.243)	−4.371 to 0.501	-	0.040 (0.095)	5.255 (4.365)	0.210 (0.529)	−0.826 to 1.247	-
Awareness	**0.792 (0.127)**	**−9.358 (0.208)**	−7.412 (1.200)	**−9.763 to 5.060**	93.0	−0.059 (0.175)	−3.883 (2.204)	0.229 (0.692)	−1.127 to 1.585	-
Encouragement	**0.193 (0.049)**	−0.455 (8.892)	−0.080 (1.716)	−3.452 to 3.276	-	0.040 (0.064)	**−17.616 (6.043)**	−0.705 (1.153)	−2.965 to 1.555	-
Rewarding	**−0.184 (0.059)**	0.526 (6.208)	−0.097 (1.143)	−2.336 to 2.143	-	**−0.200 (0.069)**	**−14.639 (5.577)**	2.928 (1.505)	−0.022 to 5.877	-
Lack of self-efficacy	**−0.383 (0.077)**	−0.426 (5.280)	0.163 (2.023)	−3.801 to 4.127		**−0.185 (0.085)**	**15.931 (4.384)**	−2.947 (1.578)	−6.041 to 0.147	-
Total	-	-	−24.212 (2.650)	**−29.405 to −19.018**	303.7	-	-	−8.814 (3.518)	**−15.709 to −1.918**	−86.6
	**Germany**					**Greece**				
	**(*n* = 1139)**					**(*n* = 1733)**				
**Parenting Practices**	**a ^†^ (SE)**	**b ^‡^ (SE)**	**ab ^§^ (SE)**	**95% CI of ab**	**% Mediated Effect ^|^**	**a ^†^ (SE)**	**b^‡^ (SE)**	**ab ^§^ (SE)**	**95% CI of ab**	**% Mediated Effect ^|^**
**Plain water**										
Availability soft drinks/prepacked FJ	**−0.366 (0.122)**	**−38.176 (6.360)**	13.972 (5.207)	**3.767 to 24.178**	23.2	**−0.492 (0.086)**	−2.838 (8.146)	1.396 (4.015)	−6.474 to 9.266	-
Availability plain water	**0.166 (0.082)**	**46.512 (7.989)**	7.721 (4.038)	−0.193 to 15.635	-	**0.193 (0.070)**	**24.297 (9.047)**	4.689 (2.438)	−0.088 to 9.467	-
Permissiveness	**−0.471 (0.111)**	−7.866 (6.881)	3.705 (3.357)	−2.874 to 10.284	-	**−0.208 (0.074)**	−13.552 (9.003)	2.819 (2.124)	−1.345 to 6.982	-
Avoiding negative modelling	**0.136 (0.069)**	0.183 (9.315)	0.025 (1.267)	−2.458 to 2.508	-	**0.146 (0.060)**	−13.490 (10.573)	−1.970 (1.743)	−5.386 to 1.447	-
Awareness	0.041 (0.114)	3.688 (5.558)	0.151 (0.478)	−0.786 to 1.089	-	**0.560 (0.104)**	−3.672 (5.955)	−2.056 (3.357)	−8.635 to 4.523	-
Encouragement	0.091 (0.049)	16.528 (13.049)	1.504 (1.437)	−1.313 to 4.321	-	0.022 (0.040)	**62.420 (16.189)**	1.373 (2.522)	−3.570 to 6.317	-
Rewarding	**−0.111 (0.052)**	**25.691 (11.269)**	−2.852 (1.830)	−6.439 to 0.735	-	**−0.114 (0.048)**	8.519 (13.636)	−0.971 (1.607)	−4.122 to 2.179	-
Lack of self-efficacy	−0.086 (0.074)	**−53.431 (8.511)**	4.595 (4.021)	−3.286 to 12.476	-	**−0.126 (0.061)**	−14.569 (10.424)	1.836 (1.586)	−1.273 to 4.944	-
Total	-	-	28.822 (7.913)	**13.313 to 44.330**	47.8	-	-	7.116 (5.552)	−3.766 to 17.998	-
**Soft drinks**										
Availability soft drinks/prepacked FJ	**−0.366 (0.122)**	**6.070 (2.338)**	−2.222 (1.132)	**−4.440 to −0.004**	8.7	**−0.492 (0.086)**	**5.234 (1.202)**	−2.575 (0.743)	**−4.032 to −1.118**	67.6
Availability plain water	**0.166 (0.082)**	−1.786 (2.930)	−0.296 (0.508)	−1.292 to 0.699	-	**0.193 (0.070)**	0.662 (1.336)	0.128 (0.262)	−0.386 to 0.641	-
Permissiveness	**−0.471 (0.111)**	**11.060 (2.530)**	−5.209 (1.711)	**−8.563 to −1.856**	20.5	**−0.208 (0.074)**	1.851 (1.328)	−0.385 (0.308)	−0.989 to 0.219	-
Avoiding negative modelling	**0.136 (0.069)**	−3.950 (3.407)	−0.537 (0.538)	−1.591 to 0.516	-	**0.146 (0.060)**	1.809 (1.561)	0.264 (0.252)	−0.231 to 0.759	-
Awareness	0.041 (0.114)	3.697 (2.036)	0.152 (0.430)	−0.691 to 0.994	-	**0.560 (0.104)**	**−3.600 (0.878)**	−2.016 (0.618)	**−3.227 to −0.805**	52.9
Encouragement	0.091 (0.049)	1.683 (4.793)	0.153 (0.444)	−0.717 to 1.023	-	0.022 (0.040)	**4.831 (2.389)**	0.106 (0.200)	−0.286 to 0.499	-
Rewarding	**−0.111 (0.052)**	**10.193 (4.130)**	−1.131 (0.701)	−2.505 to 0.242	-	**−0.114 (0.048)**	**5.227 (2.011)**	−0.596 (0.340)	−1.262 to 0.070	-
Self-efficacy	−0.086 (0.074)	0.443 (3.108)	−0.038 (0.269)	−0.566 to 0.490	-	**−0.126 (0.061)**	−0.547 (1.538)	0.069 (0.197)	−0.316 to 0.454	-
Total	-	-	−9.129 (1.899)	**−12.851 to −5.407**	35.9	-	-	−5.005 (0.942)	**−6.851 to −3.159**	131.4
**Prepacked FJ**										
Availability soft drinks/prepacked FJ	**−0.366 (0.122)**	**16.400 (3.370)**	−6.002 (2.350)	**−10.609 to −1.396**	71.1	**−0.492 (0.086)**	**18.474 (2.552)**	−9.089 (2.025)	**−13.058 to −5.120**	217.4
Availability plain water	**0.166 (0.082)**	−7.248 (4.219)	−1.203 (0.919)	−3.004 to 0.597	-	**0.193 (0.070)**	4.589 (2.883)	0.886 (0.634)	−0.357 to 2.129	-
Permissiveness	**−0.471 (0.111)**	6.426 (3.643)	−3.027 (1.858)	−6.669 to 0.165	-	**−0.208 (0.074)**	**6.686 (2.822)**	−1.391 (0.768)	−2.895 to 0.114	-
Avoiding negative modelling	**0.136 (0.069)**	−3.391 (4.899)	−0.461 (0.706)	−1.845 to 0.923	-	**0.146 (0.060)**	1.1015 (3.311)	0.148 (0.487)	−0.807 to 1.103	-
Awareness	0.041 (0.114)	**−8.335 (2.934)**	−0.342 (0.958)	−2.219 to 1.536	-	**0.560 (0.104)**	**−9.032 (1.868)**	−5.058 (1.406)	**−7.814 to −2.302**	121.0
Encouragement	0.091 (0.049)	−2.379 (6.896)	−0.216 (0.638)	−1.468 to 1.035	-	0.022 (0.040)	5.465 (5.073)	0.120 (0.245)	−0.361 to 0.601	-
Rewarding	**0.111 (0.052)**	**−15.956 (5.945)**	−1.771 (1.060)	−3.849 to 0.307	-	**−0.114 (0.048)**	−1.094 (4.274)	0.125 (0.490)	−0.836 to 1.085	-
Lack of self-efficacy	−0.086 (0.074)	7.078 (4.472)	−0.609 (0.650)	−1.882 to 0.665	-	**−0.126 (0.061)**	−0.628 (3.267)	0.079 (0.413)	−0.731 to 0.889	-
Total	-	-	−13.631 (3.089)	**−19.686 to −7.577**	161.4	-	-	−14.180 (2.381)	**−18.846 to −9.514**	339.2
	**Poland**					**Spain**				
	**(*n* = 1384)**					**(*n* = 851)**				
**Parenting Practices**	**a ^†^ (SE)**	**b ^‡^ (SE)**	**ab ^§^ (SE)**	**95% CI of ab**	**% Mediated Effect ^|^**	**a ^†^ (SE)**	**b ^‡^ (SE)**	**ab ^§^ (SE)**	**95% CI of ab**	**% Mediated Effect ^|^**
**Plain water**										
Availability soft drinks/prepacked FJ	**−0.607 (0.122)**	**−19.416 (6.719)**	11.786 (4.716)	**2.541 to 21.030**	−52.2	**−0.316 (0.119)**	**−33.204 (9.262)**	10.492 (4.917)	**0.855 to 20.130**	−72.9
Availability plain water	**0.308 (0.111)**	**57.431 (5.688)**	17.689 (6.611)	**4.731 to 30.647**	−78.4	0.051 (0.078)	**41.809 (11.830)**	2.132 (3.316)	−4.368 to 8.632	-
Permissiveness	**−0.536 (0.123)**	**−21.935 (6.478)**	11.757 (4.397)	**3.139 to 20.376**	−52.1	**−0.325 (0.128)**	5.594 (8.515)	−1.818 (2.859)	−7.421 to 3.785	-
Avoiding negative modelling	**0.418 (0.077)**	−6.202 (7.743)	−2.592 (3.272)	−9.005 to 3.820	-	0.148 (0.088)	−12.604 (10.581)	−1.865 (1.919)	−5.627 to 1.896	-
Awareness	**0.796 (0.125)**	1.435 (4.611)	1.142 (3.675)	−6.060 to 8.345	-	**0.473 (0.145)**	−11.161 (6.626)	−5.279 (3.527)	−12.193 to 1.634	-
Encouragement	**0.121 (0.058)**	**33.635 (10.731)**	4.070 (2.343)	−0.523 to 8.663	-	0.028 (0.057)	**49.545 (16.011)**	1.387 (2.859)	−4.217 to 6.992	-
Rewarding	**−0.146 (0.063)**	7.428 (8.688)	−1.084 (1.352)	−3.734 to 1.565	-	−0.033 (0.057)	28.869 (16.076)	−0.953 (1.729)	−4.341 to 2.436	-
Lack of self-efficacy	0.031 (0.082)	**−26.286 (7.033)**	−0.815 (2.166)	−5.061 to 3.431	-	−0.140 (0.082)	**−30.859 (11.138)**	4.320 (2.972)	−1.505 to 10.146	-
Total	-	-	41.952 (90.098)	**24.120 to 59.784**	−186.0	-	-	8.417 (7.826)	−6.921 to 23.755	-
**Soft drinks**										
Availability soft drinks/prepacked FJ	**−0.607 (0.122)**	**11.553 (5.164)**	−7.013 (3.437)	**−13.749 to −0.276**	12.4	**−0.316 (0.119)**	**3.963 (1.158)**	−1.252 (0.597)	**−2.422 to −0.082**	34.0
Availability plain water	**0.308 (0.111)**	**−20.685 (4.367)**	−6.371 (2.661)	**−11.587 to −1.155**	11.2	0.051 (0.078)	−1.601 (1.479)	−0.082 (0.146)	−0.368 to 0.204	-
Permissiveness	**−0.536 (0.123)**	**13.441 (4.979)**	−7.204 (3.139)	**−13.357 to −1.051**	12.7	**−0.325 (0.128)**	0.147 (1.064)	−0.048 (0.346)	−0.727 to 0.631	-
Avoiding negative modelling	**0.418 (0.077)**	−7.722 (5.958)	−3.228 (2.560)	−8.246 to 1.791	-	0.148 (0.088)	0.109 (1.324)	0.016 (0.196)	−0.368 to 0.401	-
Awareness	**0.796 (0.125)**	2.022 (3.543)	1.610 (2.832)	−56.661 to 15.324	-	**0.473 (0.145)**	−1.310 (0.829)	−0.620 (0.436)	−1.474 to 0.234	-
Encouragement	**0.121 (0.058)**	1.180 (8.245)	0.143 (1.000)	−1.817 to 2.103	-	0.028 (0.057)	−0.703 (2.002)	−0.020 (0.069)	−0.155 to 0.115	-
Rewarding	**−0.146 (0.063)**	0.868 (6.681)	−0.127 (0.977)	−2.042 to 1.788	-	−0.033 (0.057)	**4.657 (2.010)**	−0.154 (0.274)	−0.690 to 0.383	-
Lack of self-efficacy	0.031 (0.082)	**18.526 (5.388)**	0.574 (1.528)	−2.421 to 3.570	-	−0.140 (0.082)	1.740 (1.389)	−0.244 (0.241)	−0.716 to 0.229	-
Total	-	-	−21.616 (4.973)	**−31.363 to −11.869**	38.1	-	-	−2.402 (0.736)	**−3.844 to −0.960**	65.3
**Prepacked FJ**										
Availability soft drinks/prepacked FJ	**−0.607 (0.122)**	**25.879 (4.361)**	−15.709 (4.120)	**−23.784 to −7.633**	85.1	**−0.316 (0.119)**	**15.517 (3.355)**	−4.903 (2.129)	**−9.077 to −0.730**	35.9
Availability plain water	**0.308 (0.111)**	**−8.804 (3.691)**	−2.712 (1.499)	−5.650 to 0.227	-	0.051 (0.078)	7.612 (4.281)	0.388 (0.633)	−0.852 to 1.628	-
Permissiveness	**−0.536 (0.123)**	**9.648 (4.205)**	−5.171 (2.547)	**−10.164 to −0.179**	28.0	**−0.325 (0.128)**	4.072 (3.085)	−1.323 (1.130)	−3.538 to 0.891	-
Avoiding negative modelling	**0.418 (0.077)**	9.848 (5.028)	4.116 (2.234)	−0.263 to 8.496	-	0.148 (0.088)	2.492 (3.829)	0.369 (0.608)	−0.822 to 1.560	-
Awareness	**0.796 (0.125)**	**−9.656 (2.993)**	−7.686 (2.671)	**−12.921 to −2.452**	41.7	**0.473 (0.145)**	**−8.826 (2.398)**	−4.175 (1.710)	**−7.526 to −0.823**	30.5
Encouragement	**0.121 (0.058)**	2.761 (6.965)	0.334 (0.858)	−1.347 to 2.015	-	0.028 (0.057)	1.977 (5.791)	0.055 (0.197)	−0.332 to 0.442	-
Rewarding	**−0.146 (0.063)**	**−18.721 (5.641)**	2.733 (1.439)	−0.086 to 5.553	-	−0.033 (0.057)	1.356 (5.808)	−0.045 (0.207)	−0.450 to 0.360	-
Lack of self-efficacy	0.031 (0.082)	6.325 (4.559)	0.196 (0.538)	−0.858 to 1.250	-	−0.140 (0.082)	**8.454 (4.018)**	−1.184 (0.893)	−2.933 to 0.566	-
Total	-	-	−23.898 (4.961)	**−33.621 to −14.175**	129.5	-	-	−10.817 (2.770)	**−16.247 to −5.388**	79.1

SE = standard error, CI = confidence interval, FJ = fruit juice; All significant associations are presented in bold font; ^†^ a-coefficients: Associations between SES and potential mediators; ^‡^ b-coefficients: Associations between potential mediators (parenting practices) and beverage intake controlled for SES; ^§^ ab-coefficients: Mediating effect of parenting practices on the associations between SES and beverage intake; ^|^ Percentage mediated effect was not calculated if the mediation effect was not significant.
